# Combined effect of body mass index and body size perception on metabolic syndrome in South Korea: results of the fifth Korea National Health and Nutrition Examination Surveys (2010-2012)

**DOI:** 10.1186/s12889-015-1839-6

**Published:** 2015-06-17

**Authors:** Sook Hee Yoon, Kyu-Tae Han, Sun Jung Kim, Tae Yong Sohn, Byungyool Jeon, Woorim Kim, Eun-Cheol Park

**Affiliations:** Department of Health Policy and Management, Graduate School of Public Health, Yonsei University, Seoul, Republic of Korea; Department of Public Health, Graduate School, Yonsei University, Seoul, Republic of Korea; Institute of Health Services Research, Yonsei University College of Medicine, Seoul, Republic of Korea; Department of Health Administration, Namseoul University, Cheonan, Republic of Korea; Department of Health Services Administration, Yuhan University, Bucheon, Republic of Korea; Department of Preventive Medicine, Yonsei University College of Medicine, Seoul, Republic of Korea

**Keywords:** Metabolic syndrome, Body mass index, BMI, Perception of body size, Combined effect

## Abstract

**Background:**

Body mass index (BMI) has been used as an indirect predictor for the risk of metabolic syndrome. However, there are challenges in evaluating the risk of metabolic syndrome using BMI in certain parts of the world. Therefore, it is worth exploring additional factors that could supplement BMI to predict the risk of metabolic syndrome. In this study, we assessed the combined effect of BMI and perception for predicting metabolic syndrome.

**Methods:**

We used the fifth Korea National Health and Nutrition Examination Surveys (KNHANES V, 2010–12, N = 16,537) in this study. Multivariable logistic regression analysis was performed to examine the association while controlling for potential confounding variables. We also performed an analysis for the combined effect of BMI and perception of body size, and subgroup analysis by age group or moderate physical activity.

**Results:**

Data from 16,537 participants were analyzed in this study (males: 6,978, females: 9,559). Among them, metabolic syndrome was diagnosed in 1,252 (17.9 %) males and 2,445 (25.6 %) females, respectively. The combination of BMI and body size perception had a positive relation with the presence of metabolic syndrome. People who perceived themselves to be overweight for their body size had a higher risk for metabolic syndrome even if they have the same BMI.

**Conclusion:**

Our findings suggest that the combination of body size perception and BMI is useful in predicting the risk of metabolic syndrome. The use of complementary predictors could reduce the risk for inaccurate prediction of metabolic syndrome.

## Background

South Korea has achieved rapid socioeconomic development since the late 20^th^ century. This fast-paced growth has led to changes in South Koreans’ daily lives, affecting lifestyle and food consumption, and contributing to improved overall health status as South Korea becomes an aging society [1, 2]. However, there has been a concomitant increase in new health problems in South Korea, such as higher rates of chronic disease. According to Statistics Korea, cardiovascular diseases were the fifth leading cause of death in South Korea (50.2 deaths per 100,000 people in 2013) [3].

In the 2013 Organization for Economic Co-operation and Development (OECD) Health at Glance report, South Korea compares poorly with other OECD countries [4]. This problem is expected to be exacerbated by an aging population. To solve those problems, many health care professionals have studied chronic diseases and identified metabolic syndrome as a major cause [5, 6]. Metabolic syndrome has rapidly increased in South Korea over the past few decades (1998 year: 24.9 %, 2007 year: 31.3 %) [7]. Problems related with metabolic syndrome are expected to continue to increase. Thus, preventing metabolic syndrome is important for managing chronic diseases.

Metabolic syndrome is generally diagnosed by five indicators: waist circumference, triglyceride level, high-density lipoprotein (HDL) cholesterol level, blood pressure, and fasting glucose level. If three indicators (including waist circumference) are met, an individual is diagnosed with metabolic syndrome [8]. Many previous studies identified obesity as the major risk factor of metabolic syndrome. Thus, body mass index (BMI) has been widely used as an indirect predictor for evaluating the risk of metabolic syndrome [9, 10]. However, the use of BMI to predict metabolic syndrome is not necessarily applicable in every country; this simple metric does not consider important factors such as racial/ethnic differences and lifestyle factors. Even in people with the same BMI, the risk of metabolic syndrome may differ, depending on whether they smoke or consume alcohol [11–13]. Therefore, it is worth exploring additional predictors that could supplement BMI to assess the risk of metabolic syndrome; here, we focus on body size perception.

Although many previous studies have assessed the relationship between body size perception and obesity, few have also investigated the incidence of metabolic syndrome in South Korea [14, 15]. Perception of body size is a factor that affects peoples’ lifestyle, including food consumption. Moreover, the risk of metabolic syndrome can be changed by altering one’s lifestyle. In this study, we analyzed the relationship between the incidence of metabolic syndrome and BMI or body size perception, as well as the combined effect of BMI and the body size perception on metabolic syndrome.

## Methods

### Study population

This study used data from the fifth Korea National Health and Nutrition Examination Surveys (KNHANES V, 2010–12). KNHANES are cross-sectional surveys that have been conducted annually since 1998 by the Korea Centers for Disease Control and Prevention (KCDC) to assess the health and nutritional status of the Korean population. A stratified multistage cluster-sampling design was used to obtain a nationally representative sample. This survey is composed of three parts: Health Interview Survey, Health Examination, and Nutrition Survey. We used data from the Health Interview Survey, Health Examination, and Nutrition Survey. The overall response rates were 81.9 % in 2010, 80.4 % in 2011, and 80.0 % in 2012. A total of 25,967 individuals (8,958 in 2010, 8,491 in 2011, and 8,518 in 2012) completed the survey. Any respondents who did not provide BMI, perceptions of body size, five indicators for the diagnosis of metabolic syndrome, or were under the age of 19 were excluded from the study. We ultimately included 14,773 eligible participants in this study. The KNHANES was openly available in https://knhanes.cdc.go.kr/knhanes/eng/index.do after submitting e-mail address and registering short-form information. These data was approved by the KCDC Institutional Review Board, and all participants provided written informed consent (2010-02CON-21-C, 2011-02CON-06-C, 2012-01-EXP-01-2C).

### Variables

The outcome variable in this study was the incidence of metabolic syndrome, which was defined by the International Diabetes Federation (IDF) criteria. It was diagnosed if two of the five indicators (including waist circumference) met the IDF criteria for waist circumference, triglyceride level, HDL cholesterol level, blood pressure, and fasting glucose level.

*IDF criteria (metabolic syndrome diagnosed if two or more indicators were present)*

1. *Waist circumference (male: ≥90 cm and female: ≥80 cm for Asian subjects)*
2. *Triglycerides level (≥150 mg/dl)*
3. *HDL cholesterol level (male: <40 mg/dl, female: <50 mg/dl)*
4. *Blood pressure (systolic: ≥130 mmHg, diastolic: ≥85 mmHg, or treatment of diagnosed hypertension)*
5. *Fasting glucose level (≥100 mg/dl or type 2 diabetes)*

The independent variables of main interest in relation to metabolic syndrome were BMI and body size perception. BMI was calculated as body weight (kg) divided into the squared height (m^2^). BMI was classified into three groups as follows: ≤22.9, 23.0–24.9, or ≥25. Perception of body size was defined as the answer to the question: “How do you perceive your body size?” The response to this question was classified into: underweight, normal, or overweight.

Other independent variables considered in analysis as potential confounding variables were age, sex, income, educational level, economic activity, marital status, sleep duration, smoking status, alcohol consumption, stress awareness, moderate physical activity, menopause (only female), total energy intake and survey year. Income status was classified as “low”, “mid-low”, “mid-high”, or “high”. Economic activity was defined as “yes” or “no”. Stress awareness was classified as “high”, “moderate”, or “low”. Moderate physical activity was defined as whether respondents performed moderate physical activity for 30 min per session more than 5 times per week. Total energy intake was calculated based on respondent`s self-reported for their usual food consumption.

### Statistical analysis

We first examined the distribution of each categorical variable by frequency and percentages and performed *χ*^2^ tests to identify correlation with combination of BMI and body size perception by sex. Next, we performed analysis of variance (ANOVA) for continuous variables as total energy intake to identify correlation with combination of BMI and body size perception and to compare average and standard deviation of variables. In addition, these analyses were also performed to examine differences in each variable according to incidence of metabolic syndrome by sex. Multivariable logistic regression analysis was used to examine the association between BMI or body size perception and metabolic syndrome while controlling for potential confounding variables such as age, sex, income, educational level, economic activity, marital status, sleep duration, smoking status, alcohol consumption, stress awareness, moderate physical activity, menopause (only female), total energy intake, and survey year. We also included menopause status for female respondents. An additional analysis was carried out for the combined effect of BMI and body size perception, as was subgroup analysis by either age group (< vs. ≥65 years) or physical activity. Sampling weights assigned to each participant were applied in the analyses to generalize the sampled data. C-statistics were calculated to examine the predictive values for the logistics model. These values range between 0 (no predictive value) and 1, (perfect predictive value). All statistical analyses were performed using SAS statistical software (Cary, NC) version 9.2.

## Results

Data from 14,773 participants were analyzed in this study (males: 5,897, females: 8,876). Tables 1 and 2 shows the association between combination of BMI and body size perception and other covariates by sex. Among them, people who rightly perceived their body size were as follows: males = BMI, ≤22.9, 52.6 %; 23.0–24.9, 64.7 %; ≥25, 78.1 %, and females = BMI, ≤22.9, 26.2 %; 23.0–24.9, 38.0 %; ≥25, 82.5 %. There were statistically significant correlations with combination of BMI and body size perception in both sex (P < .0001). By the results of association between combination of BMI and body size perception and covariates, most of covariates had statistically significant correlations with variables of interest, except to moderate physical activity and survey year in males; moderate physical activity and menopause in females.

**Table 1:** Association between combination of BMI and perception of body size and covariates in male

	Males (n = 5,897)																		
BMI	≤22.9 (n = 2,340)						23.0–24.9 (n = 1,520)						≥25 (n = 2,037)						P-value
Perception of body size	Underweight (52.6 %)		Normal (44.0 %)		Overweight (3.3 %)		Underweight (5.3 %)		Normal (64.7 %)		Overweight (30.0 %)		Underweight (1.1 %)		Normal (20.9 %)		Overweight (78.1 %)		
Variables	N/Mean	%/SD	N/Mean	%/SD	N/Mean	%/SD	N/Mean	%/SD	N/Mean	%/SD	N/Mean	%/SD	N/Mean	%/SD	N/Mean	%/SD	N/Mean	%/SD	
Age (years)																			
19 ~ 29	119	26.6	91	20.4	14	3.1	1	0.2	51	11.4	36	8.1	0	0.0	9	2.0	126	28.2	<.0001
30 ~ 39	186	19.9	144	15.4	13	1.4	5	0.5	130	13.9	83	8.9	1	0.1	37	4.0	336	35.9	
40 ~ 49	178	17.3	136	13.2	15	1.5	10	1.0	169	16.4	79	7.7	1	0.1	72	7.0	368	35.8	
50 ~ 59	211	18.1	171	14.7	8	0.7	13	1.1	239	20.5	123	10.5	2	0.2	92	7.9	307	26.3	
60 ~ 69	247	20.4	215	17.8	13	1.1	23	1.9	216	17.9	87	7.2	10	0.8	110	9.1	289	23.9	
≥70	291	26.2	273	24.6	15	1.4	28	2.5	179	16.1	48	4.3	8	0.7	105	9.5	164	14.8	
Income																			
Low	306	27.0	235	20.7	22	1.9	31	2.7	181	15.9	57	5.0	6	0.5	106	9.3	191	16.8	<.0001
Mid-low	364	23.5	273	17.6	20	1.3	14	0.9	262	16.9	90	5.8	7	0.5	117	7.6	400	25.9	
Mid-high	295	18.3	283	17.6	18	1.1	21	1.3	276	17.2	135	8.4	6	0.4	98	6.1	477	29.6	
High	267	16.6	239	14.9	18	1.1	14	0.9	265	16.5	174	10.8	3	0.2	104	6.5	522	32.5	
Educational level																			
Below elementary school	297	24.9	262	22.0	20	1.7	34	2.9	205	17.2	59	5.0	8	0.7	125	10.5	181	15.2	<.0001
Middle school graduated	158	20.8	116	15.2	5	0.7	16	2.1	141	18.5	55	7.2	4	0.5	78	10.2	188	24.7	
High school graduated	426	21.2	352	17.6	23	1.1	14	0.7	331	16.5	162	8.1	6	0.3	128	6.4	563	28.1	
Above University graduated	351	18.1	300	15.5	30	1.5	16	0.8	307	15.8	180	9.3	4	0.2	94	4.8	658	33.9	
Economic activity																			
Yes	870	20.0	709	16.3	49	1.1	45	1.0	716	16.5	356	8.2	13	0.3	314	7.2	1,273	29.3	<.0001
No	362	23.3	321	20.7	29	1.9	35	2.3	268	17.3	100	6.4	9	0.6	111	7.2	317	20.4	
Marital status																			
Married	1,012	20.2	840	16.8	62	1.2	70	1.4	861	17.2	391	7.8	21	0.4	383	7.7	1,361	27.2	<.0001
Separated/Bereavement/Divorced	64	20.9	64	20.9	3	1.0	8	2.6	57	18.6	18	5.9	1	0.3	27	8.8	64	20.9	
Single	156	26.4	126	21.4	13	2.2	2	0.3	66	11.2	47	8.0	0	0.0	15	2.5	165	28.0	
Sleep duration																			
Less than 6 h	497	20.5	408	16.8	35	1.4	36	1.5	401	16.5	206	8.5	14	0.6	167	6.9	660	27.2	0.0025
7–8 h	644	21.0	520	17.0	36	1.2	39	1.3	517	16.9	222	7.2	6	0.2	230	7.5	852	27.8	
More than 9 h	91	22.4	102	25.1	7	1.7	5	1.2	66	16.2	28	6.9	2	0.5	28	6.9	78	19.2	
Smoking status																			
Non-smoker/Ex-smoker	704	19.2	648	17.6	49	1.3	56	1.5	635	17.3	273	7.4	17	0.5	300	8.2	992	27.0	<.0001
Smoker	528	23.8	382	17.2	29	1.3	24	1.1	349	15.7	183	8.2	5	0.2	125	5.6	598	26.9	
Alcohol consumption																			
Never	246	23.1	219	20.5	21	2.0	26	2.4	181	17.0	59	5.5	9	0.8	92	8.6	213	20.0	<.0001
Less than 1 time per month	259	23.4	197	17.8	10	0.9	13	1.2	197	17.8	81	7.3	4	0.4	63	5.7	284	25.6	
Less than 3 times per week	530	18.5	460	16.1	39	1.4	32	1.1	456	15.9	254	8.9	8	0.3	187	6.5	895	31.3	
More than 4 times per week	197	22.9	154	17.9	8	0.9	9	1.0	150	17.4	62	7.2	1	0.1	83	9.6	198	23.0	
Stress awareness																			
High	298	23.4	179	14.1	20	1.6	20	1.6	185	14.5	118	9.3	0	0.0	70	5.5	384	30.1	<.0001
Moderate	724	20.8	608	17.4	45	1.3	41	1.2	580	16.6	272	7.8	15	0.4	250	7.2	950	27.3	
Low	210	18.5	243	21.4	13	1.1	19	1.7	219	19.2	66	5.8	7	0.6	105	9.2	256	22.5	
Moderate physical activity																			
No	1,115	20.8	924	17.3	75	1.4	70	1.3	889	16.6	413	7.7	21	0.4	383	7.2	1,460	27.3	0.4056
Yes	117	21.4	106	19.4	3	0.5	10	1.8	95	17.4	43	7.9	1	0.2	42	7.7	130	23.8	
Survey year																			
2010	396	21.1	313	16.6	32	1.7	28	1.5	311	16.5	145	7.7	4	0.2	148	7.9	504	26.8	0.5417
2011	456	21.4	374	17.6	25	1.2	25	1.2	341	16.0	166	7.8	8	0.4	136	6.4	595	28.0	
2012	380	20.1	343	18.1	21	1.1	27	1.4	332	17.6	145	7.7	10	0.5	141	7.5	491	26.0	
Total energy intake	2,312.9	±906.0	2,252.5	±903.6	2,269.2	±1,033.1	2,073.1	±894.1	2,362.6	±895.1	2,321.0	±924.7	1,980.4	±713.5	2,360.0	±1,113.0	2,461.1	±963.6	<.0001
Total	1,232	20.9	1,030	17.5	78	1.3	80	1.4	984	16.7	456	7.7	22	0.4	425	7.2	1,590	27.0	

*BMI* body mass index

**Table 2:** Association between combination of BMI and perception of body size and covariates in female

	Females (n = 8,876)																		
BMI	≤22.9 (n = 4,219)						23.0–24.9 (n = 1,956)						≥25 (n = 2,701)						P-value
Perception of body size	Underweight (26.2 %)		Normal (57.6 %)		Overweight (16.3 %)		Underweight (5.7 %)		Normal (38.0 %)		Overweight (56.3 %)		Underweight (2.6 %)		Normal (14.9 %)		Overweight (82.5 %)		
Variables	N/Mean	%/SD	N/Mean	%/SD	N/Mean	%/SD	N/Mean	%/SD	N/Mean	%/SD	N/Mean	%/SD	N/Mean	%/SD	N/Mean	%/SD	N/Mean	%/SD	
Age (years)																			
19 ~ 29	135	18.1	293	39.3	116	15.5	0	0.0	15	2.0	67	9.0	0	0.0	1	0.1	119	16.0	<.0001
30 ~ 39	183	10.7	659	38.5	265	15.5	0	0.0	46	2.7	231	13.5	0	0.0	12	0.7	314	18.4	
40 ~ 49	140	8.7	515	31.9	149	9.2	2	0.1	75	4.6	286	17.7	0	0.0	18	1.1	430	26.6	
50 ~ 59	163	9.1	446	24.9	104	5.8	6	0.3	153	8.5	294	16.4	6	0.3	52	2.9	569	31.7	
60 ~ 69	181	11.8	270	17.6	31	2.0	26	1.7	227	14.8	156	10.2	20	1.3	126	8.2	494	32.3	
≥70	302	20.4	246	16.6	21	1.4	78	5.3	227	15.3	67	4.5	44	3.0	194	13.1	302	20.4	
Income																			
Low	329	17.2	364	19.0	57	3.0	70	3.7	240	12.5	128	6.7	43	2.2	195	10.2	487	25.5	<.0001
Mid-low	252	11.0	587	25.7	164	7.2	19	0.8	202	8.8	297	13.0	12	0.5	115	5.0	640	28.0	
Mid-high	232	9.9	746	31.8	236	10.1	11	0.5	148	6.3	287	12.2	9	0.4	58	2.5	618	26.4	
High	291	12.5	732	31.4	229	9.8	12	0.5	153	6.6	389	16.7	6	0.3	35	1.5	483	20.7	
Educational level																			
Below elementary school	455	15.4	492	16.7	65	2.2	103	3.5	417	14.1	229	7.8	63	2.1	314	10.6	811	27.5	<.0001
Middle school graduated	94	9.9	205	21.6	38	4.0	5	0.5	109	11.5	131	13.8	5	0.5	44	4.6	316	33.4	
High school graduated	254	9.3	833	30.4	300	11.0	4	0.1	151	5.5	420	15.3	1	0.0	33	1.2	741	27.1	
Above University graduated	301	13.4	899	40.1	283	12.6	0	0.0	66	2.9	321	14.3	1	0.0	12	0.5	360	16.0	
Economic activity																			
Yes	493	12.0	1,166	28.3	318	7.7	41	1.0	320	7.8	562	13.6	16	0.4	154	3.7	1,048	25.4	<.0001
No	611	12.8	1,263	26.5	368	7.7	71	1.5	423	8.9	539	11.3	54	1.1	249	5.2	1,180	24.8	
Marital status																			
Married	708	10.8	1,832	28.1	540	8.3	50	0.8	534	8.2	894	13.7	34	0.5	232	3.6	1,704	26.1	<.0001
Separated/Bereavement/Divorced	266	16.1	319	19.3	44	2.7	62	3.7	198	12.0	136	8.2	36	2.2	170	10.3	425	25.7	
Single	130	18.8	278	40.2	102	14.7	0	0.0	11	1.6	71	10.3	0	0.0	1	0.1	99	14.3	
Sleep duration																			
Less than 6 h	469	12.5	933	24.9	220	5.9	59	1.6	358	9.6	451	12.0	39	1.0	220	5.9	999	26.7	<.0001
7–8 h	530	12.0	1,311	29.6	398	9.0	37	0.8	328	7.4	592	13.4	23	0.5	149	3.4	1,061	24.0	
More than 9 h	105	15.0	185	26.5	68	9.7	16	2.3	57	8.2	58	8.3	8	1.1	34	4.9	168	24.0	
Smoking status																			
Non-smoker/Ex-smoker	1,043	12.3	2,312	27.3	642	7.6	109	1.3	726	8.6	1,051	12.4	69	0.8	394	4.7	2,126	25.1	0.0019
Smoker	61	15.1	117	29.0	44	10.9	3	0.7	17	4.2	50	12.4	1	0.2	9	2.2	102	25.2	
Alcohol consumption																			
Never	526	15.1	843	24.2	164	4.7	67	1.9	346	9.9	365	10.5	50	1.4	235	6.8	885	25.4	<.0001
Less than 1 time per month	367	11.3	923	28.3	287	8.8	27	0.8	252	7.7	448	13.7	15	0.5	107	3.3	833	25.6	
Less than 3 times per week	188	9.5	622	31.5	223	11.3	15	0.8	126	6.4	270	13.7	4	0.2	54	2.7	472	23.9	
More than 4 times per week	23	14.2	41	25.3	12	7.4	3	1.9	19	11.7	18	11.1	1	0.6	7	4.3	38	23.5	
Stress awareness																			
High	360	14.6	637	25.8	225	9.1	40	1.6	164	6.6	287	11.6	26	1.1	84	3.4	648	26.2	<.0001
Moderate	558	11.0	1,474	29.1	398	7.9	41	0.8	425	8.4	682	13.5	24	0.5	197	3.9	1,263	25.0	
Low	186	13.8	318	23.7	63	4.7	31	2.3	154	11.5	132	9.8	20	1.5	122	9.1	317	23.6	
Moderate physical activity																			
No	1,027	12.6	2,253	27.6	635	7.8	102	1.3	679	8.3	1,012	12.4	61	0.7	369	4.5	2,013	24.7	0.0755
Yes	77	10.6	176	24.3	51	7.0	10	1.4	64	8.8	89	12.3	9	1.2	34	4.7	215	29.7	
Survey year																			
2010	330	11.8	752	26.8	222	7.9	38	1.4	227	8.1	382	13.6	21	0.7	125	4.5	708	25.2	<.0001
2011	416	13.3	863	27.5	238	7.6	39	1.2	257	8.2	360	11.5	24	0.8	148	4.7	792	25.2	
2012	358	12.2	814	27.7	226	7.7	35	1.2	259	8.8	359	12.2	25	0.9	130	4.4	728	24.8	
Menopause																			
Not yet	464	11.1	1,499	35.8	534	12.8	3	0.1	152	3.6	599	14.3	0	0.0	34	0.8	898	21.5	0.7918
Yes	640	13.6	930	19.8	152	3.2	109	2.3	591	12.6	502	10.7	70	1.5	369	7.9	1,330	28.3	
Total energy intake	1,683.0	±649.9	1,747.8	±656.5	1,724.0	±699.4	1,447.7	±608.8	1,629.2	±576.5	1,683.4	±654.6	1,412.0	±423.2	1,610.2	±643.0	1,694.6	±650.1	<.0001
Total	1,104	12.4	2,429	27.4	686	7.7	112	1.3	743	8.4	1,101	12.4	70	0.8	403	4.5	2,228	25.1	

*BMI* body mass index

Table 3 shows the univariate associations between each variable and metabolic syndrome. Among them, metabolic syndrome was noted in 1,062 (18.0 %) males and 2,304 (26.0 %) females. In both males and females, higher BMI were more frequent in those with metabolic syndrome (males: ≤22.9, 1.5 %; 23.0–24.9, 11.1 %; ≥25, 42.1 % and females: ≤22.9, 5.3 %; 23.0–24.9, 28.5 %; ≥25, 56.4 %). By body size perception, people who responded overweight were more frequently determined to have metabolic syndrome regardless of sex (males: underweight, 2.0 %; normal, 9.9 %; overweight, 37.3 % and females: underweight, 12.7 %; normal, 18.7 %; overweight, 36.7 %). In addition, males who overestimated their body size than BMI were more frequent in those with metabolic syndrome, but females who underestimated their body size were more frequent in those with metabolic syndrome.

**Table 3:** Demographic characteristics by metabolic syndrome (frequency, %)

		Metabolic syndrome (n = 14,773)									
Variables		Males (n = 5,897)					Females (n = 8,876)				
		Yes		No		P-value	Yes		No		P-value
		N/Mean	%/SD	N/Mean	%/SD		N/Mean	%/SD	N/Mean	%/SD	
BMI											
≤22.9		36	1.5	2,304	98.5	<.0001	222	5.3	3,997	94.7	<.0001
23.0–24.9		168	11.1	1,352	88.9		558	28.5	1,398	71.5	
≥25		858	42.1	1,179	57.9		1,524	56.4	1,177	43.6	
Perception of body size											
Underweight		27	2.0	1,307	98.0	<.0001	163	12.7	1,123	87.3	<.0001
Normal		242	9.9	2,197	90.1		667	18.7	2,908	81.3	
Overweight		793	37.3	1,331	62.7		1,474	36.7	2,541	63.3	
BMI	Perception of body size										
≤22.9	Underweight	11	0.9	1,221	99.1	<.0001	63	5.7	1,041	94.3	<.0001
	Normal	20	1.9	1,010	98.1		131	5.4	2,298	94.6	
	Overweight	5	6.4	73	93.6		28	4.1	658	95.9	
23.0–24.9	Underweight	10	12.5	70	87.5		53	47.3	59	52.7	
	Normal	90	9.1	894	90.9		267	35.9	476	64.1	
	Overweight	68	14.9	388	85.1		238	21.6	863	78.4	
≥25	Underweight	6	27.3	16	72.7		47	67.1	23	32.9	
	Normal	132	31.1	293	68.9		269	66.7	134	33.3	
	Overweight	720	45.3	870	54.7		1,208	54.2	1,020	45.8	
Age (years)											
19 ~ 29		23	5.1	424	94.9	<.0001	26	3.5	720	96.5	<.0001
30 ~ 39		126	13.5	809	86.5		113	6.6	1,597	93.4	
40 ~ 49		186	18.1	842	81.9		256	15.9	1,359	84.1	
50 ~ 59		229	19.6	937	80.4		486	27.1	1,307	72.9	
60 ~ 69		275	22.7	935	77.3		689	45.0	842	55.0	
≥70		223	20.1	888	79.9		734	49.6	747	50.4	
Income											
Low		211	18.6	924	81.4	0.7869	812	42.4	1,101	57.6	<.0001
Mid-low		275	17.8	1,272	82.2		640	28.0	1,648	72.0	
Mid-high		279	17.3	1,330	82.7		471	20.1	1,874	79.9	
High		297	18.5	1,309	81.5		381	16.4	1,949	83.6	
Educational level											
Below elementary school		212	17.8	979	82.2	<.0001	1,363	46.2	1,586	53.8	<.0001
Middle school graduated		186	24.4	575	75.6		308	32.5	639	67.5	
High school graduated		337	16.8	1,668	83.2		457	16.7	2,280	83.3	
Above University graduated		327	16.9	1,613	83.1		176	7.8	2,067	92.2	
Economic activity											
Yes		761	17.5	3,584	82.5	0.0980	905	22.0	3,213	78.0	<.0001
No		301	19.4	1,251	80.6		1,399	29.4	3,359	70.6	
Marital status											
Married		959	19.2	4,042	80.8	<.0001	1,544	23.7	4,984	76.3	<.0001
Separated/Bereavement/Divorced		61	19.9	245	80.1		724	43.7	932	56.3	
Single		42	7.1	548	92.9		36	5.2	656	94.8	
Sleep duration											
Less than 6 h		439	18.1	1,985	81.9	0.9858	1,133	30.2	2,615	69.8	<.0001
7–8 h		550	17.9	2,516	82.1		973	22.0	3,456	78.0	
More than 9 h		73	17.9	334	82.1		198	28.3	501	71.7	
Smoking status											
Non-smoker/Ex-smoker		683	18.6	2,991	81.4	0.1356	2,215	26.1	6,257	73.9	0.0653
Smoker		379	17.0	1,844	83.0		89	22.0	315	78.0	
Alcohol consumption											
Never		185	17.4	881	82.6	<.0001	1,177	33.8	2,304	66.2	<.0001
Less than 1 time per month		154	13.9	954	86.1		721	22.1	2,538	77.9	
Less than 3 times per week		520	18.2	2,341	81.8		365	18.5	1,609	81.5	
More than 4 times per week		203	23.5	659	76.5		41	25.3	121	74.7	
Stress awareness											
High		221	17.3	1,053	82.7	0.0139	622	25.2	1,849	74.8	<.0001
Moderate		602	17.3	2,883	82.7		1,210	23.9	3,852	76.1	
Low		239	21.0	899	79.0		472	35.1	871	64.9	
Moderate physical activity											
No		982	18.4	4,368	81.6	0.0306	2,102	25.8	6,049	74.2	0.2222
Yes		80	14.6	467	85.4		202	27.9	523	72.1	
Survey year											
2010		360	19.1	1,521	80.9	0.0240	737	26.3	2,068	73.7	0.1602
2011		399	18.8	1,727	81.2		778	24.8	2,359	75.2	
2012		303	16.0	1,587	84.0		789	26.9	2,145	73.1	
Menopause											
Not yet		-	-	-	-	-	417	10.0	3,766	90.0	<.0001
Yes		-	-	-	-		1,887	40.2	2,806	59.8	
Total energy intake		2,365.6	±970.4	2,346.0	±935.0	0.5391	1,619.0	±640.1	1,720.2	±651.7	<.0001
Total		1,062	18.0	4,835	82.0		2,304	26.0	6,572	74.0	

*BMI* body mass index

The older age group had a higher rate of female metabolic syndrome. Notably, the distribution for metabolic syndrome had an inverse relationship with income in females (low, 42.4 %; mid-low, 28.0 %; mid-high, 20.1 %; high, 16.4 %). Similarly, subjects who were separated, widowed, or divorced were more likely to meet the criteria for metabolic syndrome compared to those with other marital statuses (males: married, 19.2 %; separated/widowed/divorced, 19.9 %; single, 7.1 % and females: married, 23.7 %; separated/widowed/divorced, 43.7 %; single, 5.2 %; Table 3).

Table 4 shows the results of logistic regression analysis for the association between BMI and metabolic syndrome adjusted for covariates by sex. In both males and females, BMI had a positive relationship with metabolic syndrome (males: ≤22.9 = ref, 23.0–24.9 odds ratio [OR]: 9.17, standard deviation [SD]: 5.81–14.50; ≥25 OR: 71.08, SD: 46.32–109.08; females: ≤22.9 = ref, 23.0–24.9 = OR: 6.79, SD: 5.57–8.28, ≥25 = OR: 27.75, SD: 22.71–33.91). Age also had a positive relationship with metabolic syndrome, whereas educational level only had an inverse relationship with metabolic syndrome in females. Both sexes who did not report economic activity had a higher risk for metabolic syndrome (males: yes = ref, no = OR: 1.50, SD = 1.10–2.05; females: no = OR: 1.27, SD = 1.08–1.48), as did smokers of males. Females who had experienced menopause had a higher risk for metabolic syndrome (not yet = ref, yes = OR: 1.46, SD = 1.09–1.94; Table 4).

**Table 4:** Results of multivariable logistic regression analysis for the relationship between BMI and metabolic syndrome

	Metabolic syndrome					
Variables	Males			Females		
	OR	SD		OR	SD	
BMI						
≤22.9	1.00	-	-	1.00	-	-
23.0–24.9	9.17	5.81	14.50	6.79	5.57	8.28
≥25	71.08	46.32	109.08	27.75	22.71	33.91
Age (years)						
19 ~ 29	1.00	-	-	1.00	-	-
30 ~ 39	2.21	1.17	4.17	2.16	1.18	3.96
40 ~ 49	3.05	1.67	5.58	4.31	2.38	7.79
50 ~ 59	4.20	2.19	8.04	5.02	2.58	9.77
60 ~ 69	5.45	2.69	11.02	8.47	4.27	16.81
≥70	7.01	3.41	14.44	12.11	6.28	23.35
Income						
Low	1.00	-	-	1.00	-	-
Mid-low	1.18	0.84	1.66	1.07	0.87	1.33
Mid-high	1.12	0.80	1.58	0.98	0.77	1.23
High	1.29	0.90	1.84	0.87	0.67	1.14
Educational level						
Below elementary school	1.00	-	-	1.00	-	-
Middle school graduated	1.25	0.90	1.74	0.71	0.56	0.91
High school graduated	0.98	0.72	1.33	0.59	0.46	0.76
Above University graduated	1.03	0.73	1.43	0.51	0.37	0.72
Economic activity						
Yes	1.00	-	-	1.00	-	-
No	1.50	1.10	2.05	1.27	1.08	1.48
Marital status						
Married	1.00	-	-	1.00	-	-
Separated/Bereavement/Divorced	0.81	0.52	1.26	1.10	0.92	1.31
Single	0.85	0.53	1.38	1.26	0.69	2.28
Sleep duration						
Less than 6 h	0.91	0.75	1.10	0.85	0.72	1.00
7–8 h	1.00	-	-	1.00	-	-
More than 9 h	0.85	0.55	1.32	1.14	0.87	1.51
Smoking status						
Non-smoker/Ex-smoker	1.00	-	-	1.00	-	-
Smoker	1.31	1.04	1.64	1.37	0.92	2.06
Alcohol consumption						
Never	1.00	-	-	1.00	-	-
Less than 1 time per month	1.03	0.73	1.46	0.96	0.80	1.16
Less than 3 times per week	1.16	0.85	1.59	1.00	0.80	1.24
More than 4 times per week	1.92	1.36	2.72	0.84	0.49	1.43
Stress awareness						
High	0.99	0.70	1.38	0.90	0.70	1.17
Moderate	0.90	0.69	1.18	0.85	0.68	1.07
Low	1.00	-	-	1.00	-	-
Moderate physical activity						
No	1.00	-	-	1.00	-	-
Yes	1.29	0.89	1.87	1.09	0.84	1.41
Survey year						
2010	1.00	-	-	1.00	-	-
2011	1.14	0.90	1.45	0.89	0.74	1.08
2012	0.82	0.63	1.07	1.14	0.95	1.37
Menopause						
Not yet	-	-	-	1.00	-	-
Yes	-	-	-	1.45	1.09	1.92
Total energy intake	1.00	0.99	1.01	1.01	0.99	1.02
C-statistics	0.855*			0.876*		

*BMI* body mass index, *OR* odds ratio, *SD*, standard deviation

*P-value for likelihood ratio test <0.05

Table 5 shows the logistic regression analysis results for the association between combined effect of BMI/body size perception and metabolic syndrome adjusted for covariates by sex. The combination of BMI and body size perception had a positive relationship with metabolic syndrome. People who perceived themselves as overweight for their body size had a higher risk for metabolic syndrome, even if they had the same BMI as a person who did not consider themselves overweight. The results of other controlling variables had similar values and trends as the results listed in Table 4 (Table 5).

**Table 5:** Results of multivariable logistic regression analysis for the relationship between BMI/body size perception and metabolic syndrome

		Metabolic syndrome					
	Variables	Males			Females		
		OR	SD		OR	SD	
BMI	Perception of body size						
≤22.9	Underweight	0.41	0.19	0.92	0.53	0.35	0.79
	Normal	1.00	-	-	1.00	-	-
	Overweight	5.38	1.42	20.36	0.98	0.59	1.65
23.0–24.9	Underweight	3.23	1.32	7.91	5.79	3.18	10.54
	Normal	5.45	2.95	10.04	5.25	3.91	7.05
	Overweight	14.85	7.75	28.45	5.89	4.41	7.85
≥25	Underweight	15.29	4.35	53.75	9.32	4.82	18.00
	Normal	25.63	13.87	47.35	18.89	12.92	27.63
	Overweight	78.80	44.44	139.72	24.50	19.20	31.26
Age (years)							
19 ~ 29		1.00	-	-	1.00	-	-
30 ~ 39		2.36	1.24	4.51	2.17	1.18	3.97
40 ~ 49		3.45	1.88	6.30	4.31	2.38	7.80
50 ~ 59		4.97	2.60	9.50	5.03	2.58	9.81
60 ~ 69		6.70	3.34	13.45	8.79	4.41	17.52
≥70		9.60	4.69	19.63	13.27	6.81	25.83
Income							
Low		1.00	-	-	1.00	-	-
Mid-low		1.16	0.82	1.64	1.06	0.85	1.32
Mid-high		1.09	0.77	1.54	0.95	0.76	1.21
High		1.23	0.86	1.77	0.86	0.65	1.12
Educational level							
Below elementary school		1.00	-	-	1.00	-	-
Middle school graduated		1.15	0.82	1.63	0.69	0.54	0.88
High school graduated		0.86	0.62	1.18	0.57	0.44	0.73
Above University graduated		0.84	0.60	1.19	0.49	0.35	0.69
Economic activity							
Yes		1.00	-	-	1.00	-	-
No		1.46	1.07	2.00	1.26	1.08	1.48
Marital status							
Married		1.00	-	-	1.00	-	-
Separated/Bereavement/Divorced		0.80	0.51	1.26	1.10	0.92	1.32
Single		0.85	0.52	1.38	1.25	0.69	2.27
Sleep duration							
Less than 6 h		0.88	0.72	1.08	0.85	0.72	1.00
7–8 h		1.00	-	-	1.00	-	-
More than 9 h		0.83	0.52	1.30	1.16	0.87	1.53
Smoking status							
Non-smoker/Ex-smoker		1.00	-	-	1.00	-	-
Smoker		1.31	1.05	1.65	1.37	0.91	2.06
Alcohol consumption							
Never		1.00	-	-	1.00	-	-
Less than 1 time per month		1.01	0.71	1.43	0.95	0.79	1.14
Less than 3 times per week		1.12	0.81	1.54	0.98	0.79	1.22
More than 4 times per week		1.91	1.33	2.73	0.83	0.48	1.41
Stress awareness							
High		0.93	0.66	1.31	0.90	0.70	1.17
Moderate		0.87	0.66	1.15	0.85	0.68	1.06
Low		1.00	-	-	1.00	-	-
Moderate physical activity							
Yes		1.00	-	-	1.00	-	-
No		1.25	0.87	1.80	1.09	0.84	1.42
Survey year							
2010		1.00	-	-	1.00	-	-
2011		1.12	0.87	1.43	0.90	0.74	1.09
2012		0.84	0.64	1.10	1.15	0.95	1.38
Menopause							
Not yet		-	-	-	1.00	-	-
Yes		-	-	-	1.46	1.09	1.94
Total energy intake		1.00	0.99	1.01	1.01	0.99	1.02
C-statistics		0.865*			0.877*		

*BMI* body mass index, *OR* odds ratio, *SD* standard deviation

*P-value for likelihood ratio test <0.05

We also performed subgroup analysis for the combined effect of BMI/body size perception by age group (< vs. ≥65 years) or moderate physical activity to identify possible differences in each group. In the subgroup analysis by age group, it revealed similar relationships of the combined effect of BMI and body size perception in these two groups as were observed in the overall analysis. However, there were some notable findings in non-elderly females. In the overweight group based on BMI, the risk for metabolic syndrome was inversely associated with body size perception (Table 6). In the results of subgroup analysis by moderate physical activity, overweight or obese people based on BMI who underestimated their body size had a higher trend regarding the risk of metabolic syndrome in the moderate physical activity of over 5 times per week group than the other group (data not shown). In the overall multivariable logistic regression, C-statistics were higher in the combination model of BMI and body size perception than when using only BMI models.

**Table 6:** Results of subgroup analysis for the relationship between combined effect of BMI/body size perception and metabolic syndrome by age group

		Metabolic syndrome											
Type of predictor for metabolic syndrome		Males						Females					
		Less than 64 years			More than 65 years			Less than 64 years			More than 65 years		
		OR	SD		OR	SD		OR	SD		OR	SD	
BMI	Perception of body size												
≤22.9	-	1.00	-	-	1.00	-	-	1.00	-	-	1.00	-	-
23.0–24.9	-	9.13	4.87	17.12	10.74	6.00	19.21	9.13	6.46	12.88	5.32	4.04	7.02
≥25	-	74.63	42.15	132.16	55.07	30.98	97.88	39.26	28.46	54.15	15.78	11.77	21.15
≤22.9	Underweight	0.39	0.12	1.28	0.41	0.13	1.32	0.47	0.19	1.13	0.40	0.25	0.64
	Normal	1.00	-	-	1.00	-	-	1.00	-	-	1.00	-	-
	Overweight	5.29	1.04	26.88	2.46	0.43	14.13	1.15	0.60	2.20	1.25	0.47	3.38
23.0-24.9	Underweight	0.97	0.17	5.73	6.10	1.97	18.95	21.35	5.80	78.56	2.75	1.58	4.80
	Normal	4.81	1.95	11.89	6.97	3.36	14.46	7.02	4.37	11.27	3.54	2.36	5.31
	Overweight	15.32	6.06	38.74	12.20	4.77	31.22	7.96	5.13	12.37	4.44	2.59	7.60
≥25	Underweight	23.41	3.74	146.52	7.82	1.38	44.43	10.18	2.27	45.72	6.52	3.14	13.53
	Normal	26.09	10.87	62.63	25.64	11.60	56.68	24.44	13.40	44.56	13.81	8.50	22.44
	Overweight	75.93	33.14	173.96	59.06	28.24	123.50	36.60	24.83	53.95	10.13	6.97	14.73
C-statistics	Only BMI Model	0.846*			0.856*			0.877*			0.784*		
	Combination Model	0.858*			0.864*			0.882*			0.791*		

*P-value for likelihood ratio test <0.05

## Discussion

Due to the rapidly aging population, it is expected that the prevalence of metabolic syndrome will continue to increase in South Korea [16]. It is therefore necessary to design effective strategies to prevent and manage this chronic condition. In recent years, BMI has become a widely used indicator of obesity and indirect predictor for metabolic syndrome. However, it had some limitations that were not overall considered to risk factors for metabolic syndrome [17, 18]. Thus, it is necessary to find complementary predictive factors; we focused on body size perception as a novel predictor for evaluating metabolic syndrome risk. Our results suggest that metabolic syndrome risk was positively related with BMI and were similar to previous studies that examined metabolic syndrome risk factors.

In addition, we observed a combined effect of body size perception and BMI on the risk of metabolic syndrome. Notably, the risk was clearer than that observed using BMI only, and was even observed in subjects with the same BMI but different body perceptions.

In predicting risk for chronic diseases as metabolic syndrome, using only BMI could make some misidentifications because it was calculated by just considering height and weight. If people had same BMI, the risk for metabolic syndrome could be different by major factors consisted of body constitution such as muscle mass and higher body fat [19]. Therefore, using combination of BMI and body size perception would be more helpful in predicting for risk. Based on our results, perception of body size as overweight had higher risk for metabolic syndrome. This is because that perception of body size as overweight could more reflect to risk for metabolic syndrome considering actual body image in same BMI. Perception of body size can help role of complementation of predicting for metabolic syndrome [20]. Therefore, it is suggested that people who perceive their body size as overweight are likely to be at risk of metabolic syndrome. In another point of view, people could respond as overweight for their body size due to their unhealthy behaviors such as unhealthy diet and insufficient physical activity for preventing chronic diseases even if people with same BMI and similar body constitution [21]. Therefore, perception of body size could be indirect indicators for reflecting life styles as well as actual body image.

The same phenomenon was observed when we performed a subgroup analysis by age group that excluded females who were overweight based on BMI and <65 years. This relationship was more positive in males, while the different results in females <65 years may be caused by younger females who did not exhibit health behaviors such as wrong diet and insufficient exercise due to their misperception for their body size despite being overweight or obesity based on BMI. However, in the case of elderly females, they had an effort to manage their health status due to their health concern by advanced age [22]. Based on the results of the subgroup analysis in the moderate physical activity group, people overweight or obese based on BMI tend to exhibit unhealthy behaviors by underestimating their body size and risks of gaining metabolic syndrome as they show moderate physical activity. They may be overconfident, believing in an improvement of their health status by sufficient physical activity, and could take more risky behaviors such as excessive eating. Therefore, providing correct information about preventing metabolic syndrome would be needed.

Although more detailed studies are needed, our findings suggest that inappropriate perception of their health status could be caused to unhealthy behaviors at risky population. This has been described previously; people who are borderline for chronic disease risk do not usually feel that their lives are at risk [23]. Conversely, high-risk populations were much more amenable to health behaviors to modify their risk. It is important to note that males tend to evaluate their own body status more favorably than females. Perception differences can induce people to make lifestyle changes (e.g., food or alcohol consumption, exercise, smoking status, etc.) [15, 24, 25].

In accordance with this, we found that South Korean subjects with the same BMI exhibited different behaviors based on their body size perception; therefore, predicting metabolic syndrome risk solely based on BMI did not take different behaviors into account [26, 27].

Thus, our findings suggest that the combination of body size perception and BMI could be more useful in predicting the risk of metabolic syndrome than BMI alone. The use of complementary predictors could improve prediction and prognostication.

This study has several strengths compared to previous investigations. First, we used nationally representative data, so our study results are representative and generalizable to South Korea citizens. Such data are especially helpful in establishing evidence-based health policies. To our knowledge, this is the first attempt to study the relationship between the combined effect of BMI/body size perception and metabolic syndrome in South Korea, despite numerous issues regarding the management of these health issues in the country. Therefore, our findings should be helpful in identifying ways to address these critical issues.

Our study also has some limitations. First, due to the cross-sectional nature of the KNHANES, it is not possible to identify causal relationships. Other issues must be considered to more accurately measure the relationship between the combined effect of BMI/body size perception and metabolic syndrome. Next, our findings included high OR values, not general OR values. Further studies are needed to confirm our findings, which show a combined effect for metabolic syndrome in relatively small study populations (after stratification). Nevertheless, the overall trends of our findings have serious implications for the management of metabolic syndrome. Third, body size perception was measured by the subjects’ answers to the question: “How do you perceive your body size?” The response could have been incorrectly perceived by researchers and is not a truly scientific measurement. Finally, our analysis did not include important details such as respondent food consumption. Thus, multiple variables that are not a major factor of metabolic syndrome were not considered in our findings.

Despite these limitations, our findings suggest that the combined effect of BMI and body size perception can be used to predict the presence of metabolic syndrome. Based on these findings, it is important for health policy makers to identify solutions for controlling metabolic syndrome. However, further studies of those issues are needed to establish an effective strategy.

## Conclusion

The combined effects of body size perception and BMI affect the risk for metabolic syndrome in individuals with the same BMI. Our findings suggest that both variables should be used in predicting the risk of disease to reduce risk of inaccurate predictions.

## Abbreviations

OECD: Organization for Economic Co-operation and Development
HDL: High-density lipoprotein
BMI: Body mass index
KNHANES: Korea National Health and Nutrition Examination Surveys
KCDC: Korea Centers for Disease Control and Prevention
IDF: International Diabetes Federation
OR: Odds ratio
SD: Standard deviation
